# Supplementary material for: Prognostic Impact of the AML60+ Score for Elderly Patients with Acute Myeloid Leukemia Treated with Hypomethylating Agents: A Retrospective Multicentric Analysis
Source: Cancers (Basel). 2025 Aug 14;17(16):2658. doi: 10.3390/cancers17162658 (PMC12384718; doi:10.3390/cancers17162658)

Supplementary methods:

Next generation sequencing

DNA was isolated from bone marrow aspirate or peripheral blood samples by automated DNA purification on a Maxwell® RSC instrument (Promega, Fitchburg, WI, USA). DNA concentration was measured with the Qubit™ dsDNA BR (Broad Range) Assay Kit and the Qubit Flex Fluorometer instrument (Thermo Fisher Scientific, San Francisco, CA, USA). For NGS-based mutation analysis, the SOPHiA DDM Myeloid Solution (MYS) panel from SOPHiA GENETICS was used that targets all exonic regions of 9 genes and exonic hotspots of 21 genes (SOPHiA GENETICS SA, Rolle, VD, Switzerland). MYS libraries were prepared manually according to the manufacturer's instructions. Briefly, individually barcoded libraries were generated from 200 ng of sample genomic DNA. Targets were enriched using a hybridization-capture based approach after equimolar pooling of the libraries. The final library was quantified with Qubit dsDNA 1X Assay and diluted for paired-end sequencing on an Illumina MiniSeq instrument (2 x 150bp; Illumina, San Diego, CA, USA). The data analysis was performed on the generated FASTQ sequencing files using the SOPHiA DDM platform (DDM version: 6.5.2--h458640-02a1edc, reference genome GRCh37 [hg19], minimum cutoff variant allele frequency: 2.5%). Variant interpretation was done according to the AMP-/ACMG-guidelines (PMID: 27993330, 35101336).

Supplementary Table S1A: Components of the AML60+ (ref 18).

**Variable**	**Points**
TP53 mutation	3
Monosomal karyotype	3
Age >65 years	2
Male sex	1
White blood cell count >20x10^9/l	1
ASXL1 mutation	1
DNMT3A mutation	1
FLT3-ITD mutation	1
RUNX1 mutation	1

Supplementary Table S1B: Risk groups according to AML60+ (ref 18)

**Points**	**Risk group**
0-1	Favorable
2-3	Intermediate
4-5	Poor
≥6	Very poor

Supplementary Table S2A: Distribution of risk factors according to ELN2022

**ELN2022**	**Classifying risk factor**	**n**	**(%)**
favorable	Mutated NPM1 without *FLT3*-ITD	10	8.5
intermediate	Mutated NPM1 with *FLT3*-ITD	3	2.6
	t(9;11)(p21.3;q23.3)	1	0.9
	Wild-type NPM1 with *FLT3*-ITD	1	0.9
	Cytogenetic and/or molecular abnormalities not classified as favorable or adverse*	11	9.4
adverse	complex karyotype and *TP53* wildtype	3	2.6
	complex karyotype and mutated *TP53*	8	6.8
	monosomale karyotype and *TP53* wildtype	8	6.8
	monosomale karyotype and mutated *TP53*	20	17.1
	del(5q)	3	2.6
	inv(3)	1	0.9
	t(v;11q23.3)/*KMT2A*-rearranged	2	1.7
	Adverse risk due to mutation profile (with intermediate risk cytogentics):		
	ASXL1	4	3.4
	ASXL1, BCOR, RUNX1	1	0.9
	ASXL1, RUNX1	1	0.9
	ASXL1, RUNX1, SRSF2, STAG2	1	0.9
	ASXL1, RUNX1, STAG2	1	0.9
	ASXL1, SRSF2	6	5.1
	ASXL1, SRSF2, STAG2	1	0.9
	ASXL1, U2AF1	3	2.6
	BCOR	2	1.7
	BCOR, EZH2	1	0.9
	BCOR1, RUNX1, STAG2	1	0.9
	EZH2	2	1.7
	RUNX1	5	4.3
	RUNX1, SF3B1	2	1.7
	RUNX1, SRSF2	1	0.9
	SRSF2	3	2.6
	SRSF2, RUNX1	1	0.9
	SRSF2, STAG2	2	1.7
	STAG2	2	1.7
	TP53	6	5.1
	Total	117	100.0

*For details: please see Table S2B

Supplementary Table S2B: Cytogenetic and/or molecular abnormalities not classified as favorable or adverse according to ELN2022 found in patients with intermediate risk according to ELN2022

**Cytogenetic aberrations and mutations**	**n**	**%***
t(2,6)(p2?5;p2?1), no mutation	1	0.9
Trisomy 8, mutated FLT-3 (non-ITD) and IDH2	1	0.9
Trisomy 11 and mutated DNMT3A and IDH1	1	0.9
Normal karyotype and mutated DDX41	1	0.9
Normal karyotype and mutated IDH1	1	0.9
Normal karyotype and mutated IDH2	1	0.9
Normal karyotype and mutated TET2	1	0.9
Normal karyotype and mutated IDH1 and KIT	1	0.9
Normal karyotype and mutated TET2 and CEBPA	1	0.9
Normal Karyotype and no mutation detected	2	1.7
Total	11	9.4

* relative to the total cohort (n=117)

Supplementary Table S3: Distribution of risk factors according to mPRS (n=121)

mPRS class	Risk factor	n	%
lower benefit (n=35)	TP53	35	28.9
intermediate benefit (n=18)	FLT3-ITD	4	3.3
	KRAS	1	0.8
	KRAS and NRAS	1	0.8
	NRAS	10	8.2
	NRAS and FLT3-ITD	2	1.7
higher benefit (n=68)*	DNMT3A	19	15.7
	IDH2	18	14.8
	SRSF2	18	14.8
	ASXL1	17	14.0
	TET2	16	13.2
	IDH1	14	11.5
	NPM1	13	10.7
	RUNX1	13	10.7
	STAG2	9	7.4
	U2AF1	6	4.9
	CEBPA	5	4.1
	EZH2	5	4.1
	BCOR	4	3.3
	FLT3-non-ITD	4	3.3
	NF1	4	3.3
	BCORL	3	2.5
	JAK2	3	2.5
	ETV6	2	1.7
	GATA2	2	1.7
	PTPN11	2	1.7
	SETBP1	2	1.7
	SF3B1	2	1.7
	WT1	2	1.7
	BRAF	1	0.8
	CBL	1	0.8
	DDX41	1	0.8
	KDM6A	1	0.8
	KIT	1	0.8
	MPL	1	0.8
	RAD21	1	0.8
	No mutation detected	6	7.9

*several mutations per case possible

Supplementary Table S4: Frequency of risk factors in the single risk groups according to AML60+

	Whole cort (n=105)		AML60+ Favorable (n=2)		AML60+ Intermediate (n=34)		AML60+ Poor (n=36)		AML60+ Very poor (n=33)	
**Risk factor**	**n**	**%**	**n**	**%**	**n**	**%**	**n**	**%**	**n**	**%**
TP53 mutation	30	28.5	0	0	1	2.9	4	11.1	25	75.8
Monosomal karyotype	25	23.8	0	0	0	0	0	0	25	75.8
Age >65 years	100	95.2	0	0	32	94.1	36	100	32	97.0
Male sex	55	52.4	2	100	14	41.2	21	58.3	18	54.5
White blood cell count >20x10^{9}/l	25	23.8	0	0	4	11.8	16	44.4	5	15.2
ASXL1 mutation	23	21.9	0	0	1	2.9	18	50.0	4	12.1
DNMT3A mutation	17	16.2	0	0	5	14.7	9	25	3	9.1
FLT3-ITD mutation	4	3.8	0	0	1	2.9	2	5.6	1	3
RUNX1 mutation	15	14.3	0	0	2	5.9	11	30.6	2	3.1

Supplementary Table S5 *p*-values for the pairwise comparisons between the categories according to ELN2002 using the log-rank test for patients treated with HMA without venetoclax.

ELN2022			mPRS			AML60+		
	**adverse**	**intermediate**		**lower benefit**	**intermediate benefit**		**very poor**	**poor**
**intermediate**	0.499	NA	**intermediate benefit**	0.831	NA	**poor**	0.427	NA
**favorable**	0.871	0.729	**higher benefit**	0.489	0.489	**intermediate/ favorable**	**0.020**	**0.020**

p-values were corrected for multiple testing using the Benjamini-Hochberg correction.

Supplementary Table S6 Univariable Cox regression models for OS by ELN2022, mPRS and AML60+ for patients treated with HMA without Venetoclax

	**HR**	**95% CI**	**p**
**ELN2022**			
Adverse*	--	--	
Intermediate	0.36	0.08, 1.56	0.2
Favorable	0.89	0.27, 2.95	0.9
**mPRS**			
Lower benefit*	--	--	
Intermediate benefit	1.09	0.33, 3.56	0.9
Higher benefit	0.62	0.29, 1.33	0.2
**AML60+**			
Very poor*	--	--	--
Poor	0.77	0.36, 1.67	0.5
Intermediate/favorable	0.25	0.09, 0.70	**0.008**

Supplementary Figure S1:

A, Boxplot of age; B, Univariable Cox regression model for OS by log(Age); C, Schoenfeld residuals for the Cox model for OS by log(Age)

A

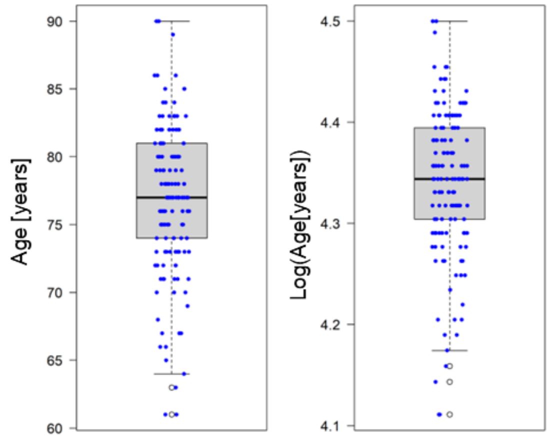

Boxplot of Age

C

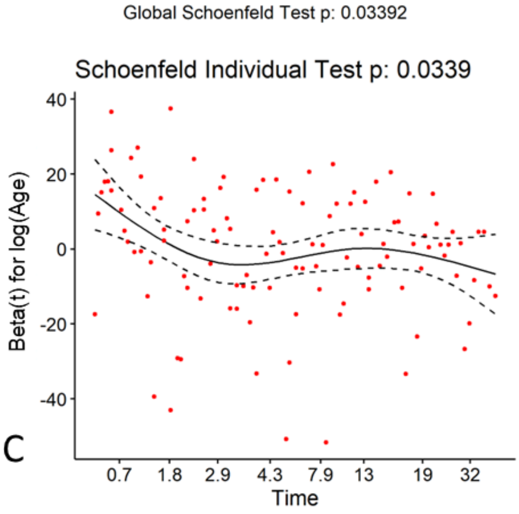

Schoenfeld residuals for the cox model for Overall Survival by log(Age)

B

	HR	95% CI	p-value
log(Age)	0.88	0.06, 1.8	>0.9

Univariable Cox regression model for Overall Survival by log(Age) as a continuous variable

HR=Hazard ratio, CI= Confidence interval

Supplementary Figure S2 :

A, Boxplot of Leukocyte count at diagnosis; B, Univariable Cox regression model for OS by log(White blood cell count at diagnosis); C, Schoenfeld residuals for the Cox model for OS by log(White blood cell count at diagnosis).

A

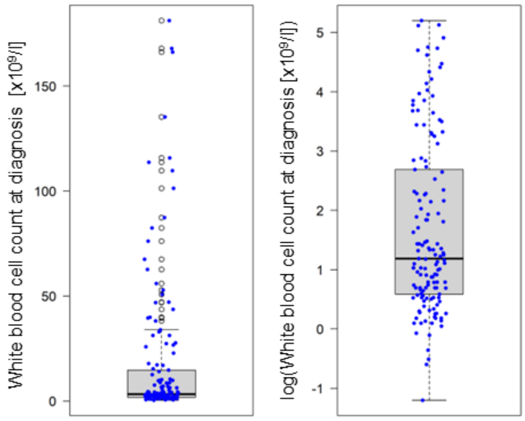

Boxplot of White blood cell count at diagnosis

C

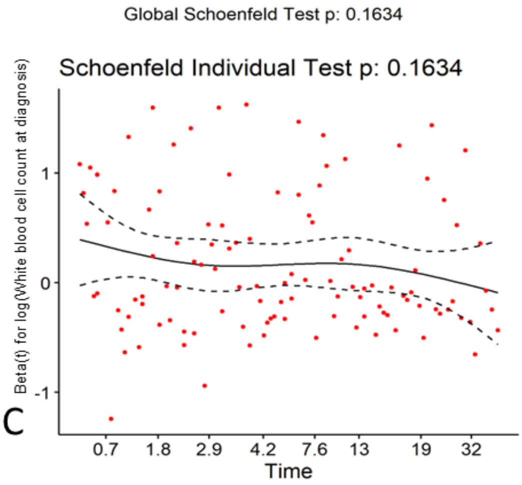

Schoenfeld residuals for the Cox model for Overall Survival by log(White blood cell count at diagnosis)

B

	HR	95% CI	p-value
log(White blood cell count at diagnosis)	1.18	1.05, 1.34	0.008

Univariable Cox regression model for Overall Survival by log(White blood cell count at diagnosis)
HR=Hazard Ratio, CI= Confidence Interval

Supplementary Figure S3. Kaplan-Meier estimates for overall survival for patients with available AML60+ according to the mutational status of *IDH2*.

IDH2-Status 0 = *IDH2* wildtype or *IDH2*-mutations with concomitant mutations in *K/N-RAS, FLT3*-ITD or *TP53*.

IDH2 Status 1= *IDH2*-mutation without concomitant mutations in *K/N-RAS, FLT3*-ITD or *TP53*.

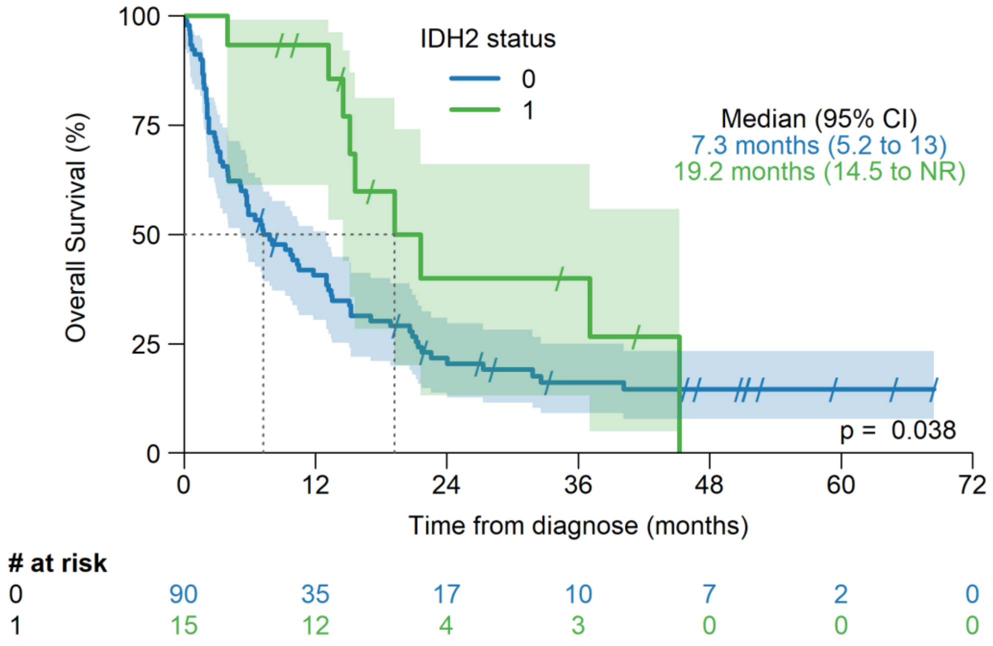

Supplementary Figure S4. Kaplan-Meier estimates for overall survival for patients with available AML60+ according to the mutational status of *NPM1*

NPM1-Status 0 = *NPM1* wildtype or *NPM1*-mutations with concomitant mutations in *K/N-RAS*, *FLT3*-ITD or *TP53*.

NPM1-Status 1 =*NPM1*-mutation without concomitant mutations in *K/N-RAS*, *FLT3*-ITD or *TP53*.

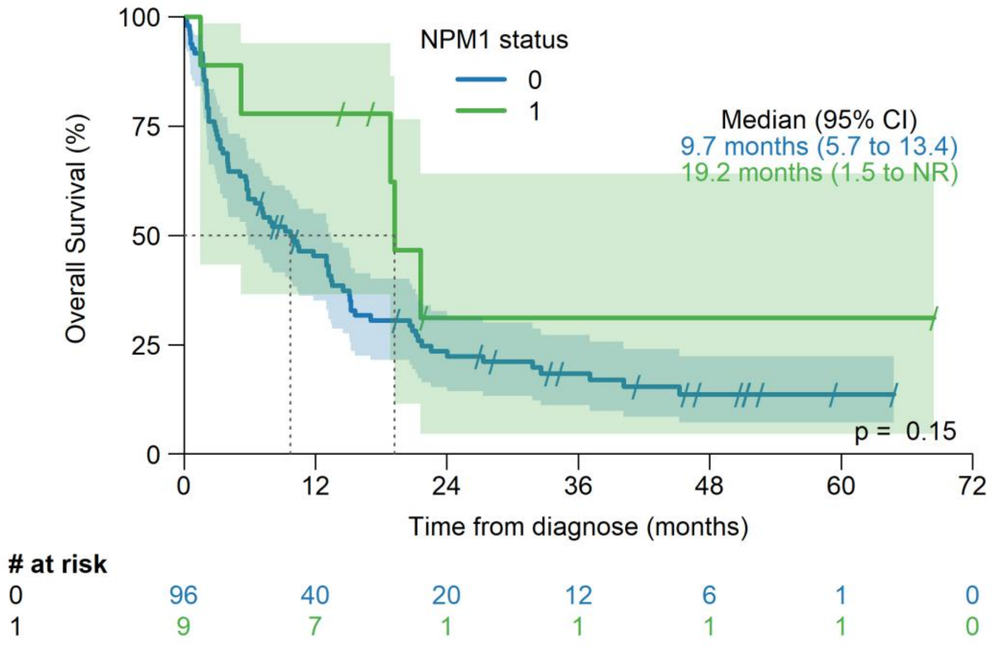

Supplementary Figure S5 Kaplan-Meier estimates for overall survival for patients treated with HMA without Ven by ELN2022, n=36 (A), AML60+, n=35 (B) and mPRS, n=38 (C)

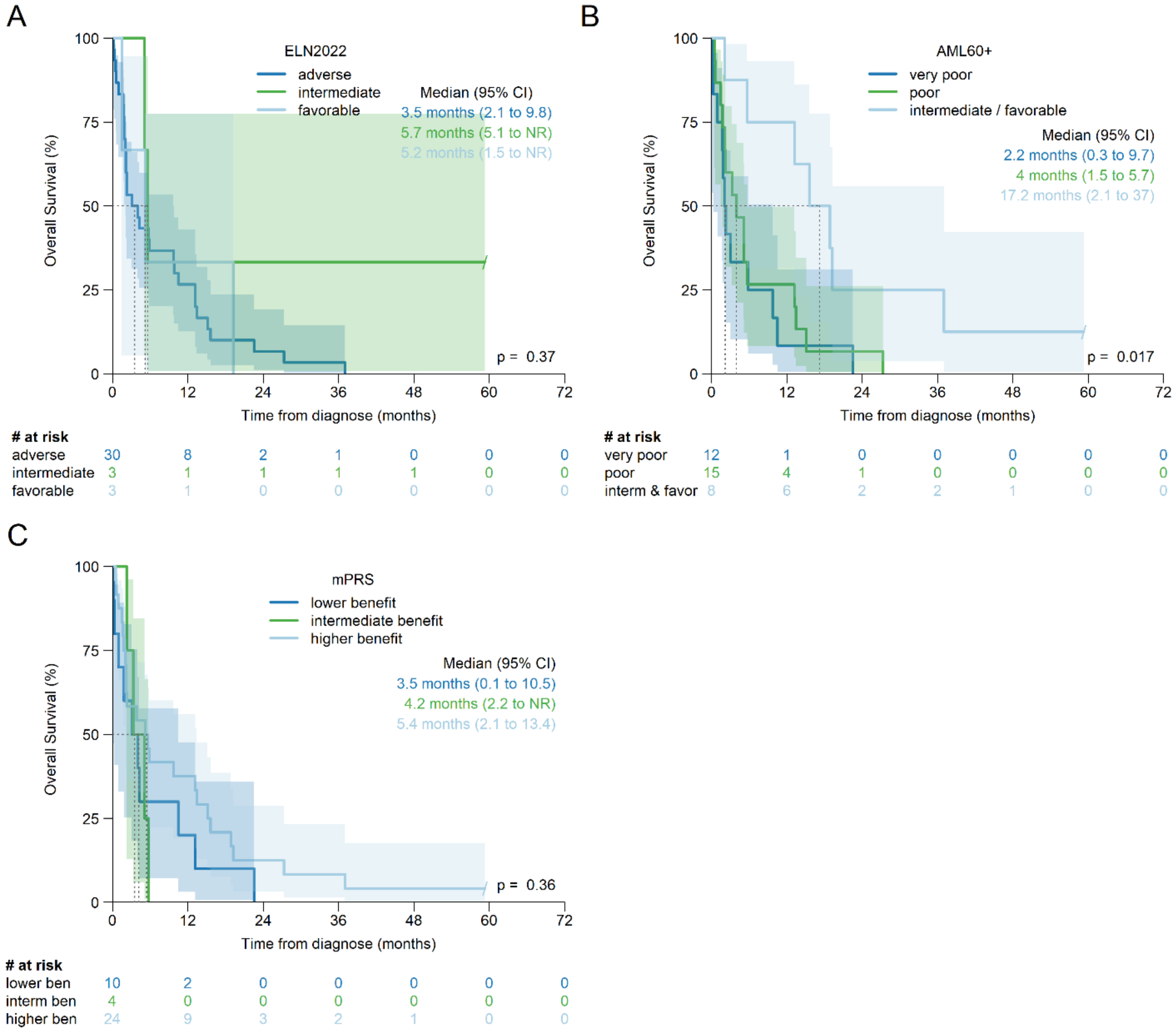

Supplement: Supplementary file 1 [file cancers-17-02658-s001.zip › cancers-3757869-supplementary.pdf]
